# The illusion of absence: how a common feature of magic shows can explain a class of road accidents

**DOI:** 10.1186/s41235-021-00287-0

**Published:** 2021-03-24

**Authors:** Vebjørn Ekroll, Mats Svalebjørg, Angelo Pirrone, Gisela Böhm, Sebastian Jentschke, Rob van Lier, Johan Wagemans, Alena Høye

**Affiliations:** 1 Department of Psychosocial Science, University of Bergen, Postboks 7807, 5020 Bergen, Norway; 2 Donders Institute for Brain, Cognition and Behaviour, Radboud University, Nijmegen, The Netherlands; 3 Department of Brain & Cognition, University of Leuven, Leuven, Belgium; 4 Department of Safety and Security, Institute of Transport Economics, Oslo, Norway

**Keywords:** Perception, Road safety, Magic, A-pillar obstruction, Blind zone, Illusion of absence, Amodal completion, LBFTS accidents, Improper lookout, View obstructions

## Abstract

The purpose of the present note is to draw attention to the potential role of a recently discovered visual illusion in creating traffic accidents. The illusion consists in a compelling and immediate experience that the space behind an occluding object in the foreground is empty. Although the illusion refers to a region of space, which is invisible due to occlusion (a blind spot), there is evidence to suggest that it is nevertheless driven by visual mechanisms and that it can be just as deceptive and powerful as ordinary visual illusions. We suggest that this novel illusion can make situations involving blind spots in a road user's field of view even more dangerous than one would expect based on the lack of visibility by itself. This could be because it erroneously makes the road user feel that (s)he has actually seen everything there is to see, and thus has verified that the blind spot is empty. This hypothesis requires further testing before definitive conclusions can be drawn, but we wish to make researchers and authorities involved in the analysis of traffic accidents and on-the-spot crash investigations aware of its potential role in order to encourage registration of relevant data and facilitate further research.

## Significance statement

Recent research suggests that the surprise and experience of impossibility experienced when things appear to materialize out of thin air in magic shows is often in part due to a previously unknown visual illusion we refer to as “the illusion of absence”. Here, we spell out how this illusion may be relevant for our understanding of traffic accidents involving blind zones, such as those created by the roof supports next to the windshield in cars. We review preliminary evidence from basic vision research suggesting the illusion of absence may render drivers “mentally blind” to the perils of certain blind zones, thus inhibiting appropriate caution and heightening the risk of traffic accidents. We argue that more basic research into the critical stimulus conditions triggering the illusion of absence may have important implications for evaluating the relative effectiveness of different countermeasures against accidents involving blind zones. Further research on the illusion of absence may also have important implications for the legal questions pertaining to driver negligence and culpability. Awareness of the potential role of this novel and counterintuitive illusion may also guide future applied research in road accident analysis and prevention.

## Introduction

“In road traffic with a considerable physical and human inertia it is obvious that failure to detect the other road user early enough is a main source of error. This conclusion is also supported by explanations of their traffic accidents which people give in court. The most frequent explanations for such accidents is “I saw him too late”, “Suddenly he was there”, etc.”

- Rumar (1990, p. 1285)

“Accidents do not occur because people gamble and lose, they occur because people do not believe that the accident about to occur is at all possible.”

- Wagenaar and Groeneweg (1987, p. 596)

Seeing something suddenly appear out of thin air can be an awe-inspiring and pleasurable experience when enjoyed in the context of a magic show (Kuhn, 2019; Leddington, 2016). A pedestrian, cyclist, motorcyclist or car appearing out of thin air in right in front of the vehicle you are driving would be equally mysterious, but obviously deeply traumatic, rather than enjoyable.

Of course, things never appear out of thin air, neither in magic shows, nor in everyday life, but they do *seem* to appear out of thin air in magic shows, and car drivers involved in an accident often report that another road user *seemed* to appear out of nowhere just before impact (Green, 2018; Marshall et al., 2012; National Academies of Sciences, Engineering, and Medicine, 2008; Phillips et al., 1990; Rumar, 1990). Because questions of culpability are involved, such statements made by car drivers may not always reflect the actual state of affairs, but we cannot a priori rule out the possibility that at least some of these reports are accurate descriptions of the driver's experience of the situation. Given that things seem to appear out of nowhere both in magic shows and in the context of road accidents, it appears plausible that considering when and why this happens in the former case may shed light on when and why it happens in the latter case.

One way in which something may seem to appear out of nowhere is already reasonably well understood both in basic cognitive science and in road safety research. Due to the counter-intuitive and powerful phenomenon of inattentional blindness (Koivisto et al., 2004; Kuhn & Tatler, 2011; Mack & Rock, 1998; Macknik et al., 2008; Most & Astur, 2007; Simons & Chabris, 1999; Triesch et al., 2003), a driver may fail to notice another road user before impact or before it is too late to take appropriate action. There is good reason to believe that inattentional blindness is an important factor in the large number of accidents categorized as “looked-but-failed-to-see” (LBFTS) or “sorry-mate-I-did-not see-you” (SMIDSY) accidents (Brown, 2002; Crundall et al., 2012; Green, 2018; Hills, 1980; Pammer et al., 2018; Sabey & Staughton, 1975; Sagberg & Sundfør, 2016; Sagberg et al., 2016; White, 2006). The reason why the other road user may seem to appear “out of nowhere” in accidents involving inattentional blindness is not the inattentional blindness in itself, but rather its counter-intuitive nature. Due to a pervasive and well-known failure of visual metacognition (Levin, 2002), we are, as it were, blind to our own inattentional blindness. Another road user may be located in a region of the visual field where we are effectively blind due to inattentional blindness, yet we may at the same time have the misleading impression that we have a good view of this region of the visual field. Thus, when the other road user moves out of this “attentional blind zone”, (s)he will seem to have appeared out of nowhere.

Another interesting way in which something may seem to appear out of nowhere has only recently been described in cognitive science (Ekroll et al., 2017; Svalebjørg et al., 2020; Øhrn et al., 2019). When an object seems to appear out of thin air in a magic show, it is often produced from a nearby hiding place, such as behind the magician's thumb or palm (Ekroll et al., 2017). But when the spectators try to figure out what just happened, they almost invariably fail to consider this rather mundane and nominally obvious possibility and instead have the impression that something impossible (i.e. magical) just happened (Svalebjørg et al., 2020). Why are people so easily and consistently fooled by such simple tricks? Ekroll et al. (2017) have proposed that this is because they are victims of a previously unknown and highly counter-intuitive visual illusion, which makes them immediately and automatically experience the objectively invisible space behind an occluder (such as the magician’s thumb) as empty, although the soon-to-appear object is actually hidden in it. When the object is pulled out from this perceptually empty space, it seems to materialize out of nowhere. The purpose of the present paper is to give an overview of what we already know about this novel visual illusion and illustrate how it may be an important factor in LBFTS-type traffic accidents. At present, we cannot draw definitive conclusions about the latter, but we hope to make clear that there are compelling arguments suggesting that this illusion of absence may play a pervasive and important role in traffic safety, and thereby encourage further research on this issue both in basic cognitive science and road safety research. We regard this as particularly important due to the counter-intuitive nature of the illusion of absence, which may not only make it particularly deceptive and hazardous, but also difficult to even imagine for investigators and researchers analysing road accidents. Ultimately, we envision that this research may have important implications for vehicle and roadway design, as well as for legal questions of culpability and negligence.

## The illusion of absence and our preliminary scientific understanding of it

### Informal demonstrations of the illusion of absence

**Fig. 1:**
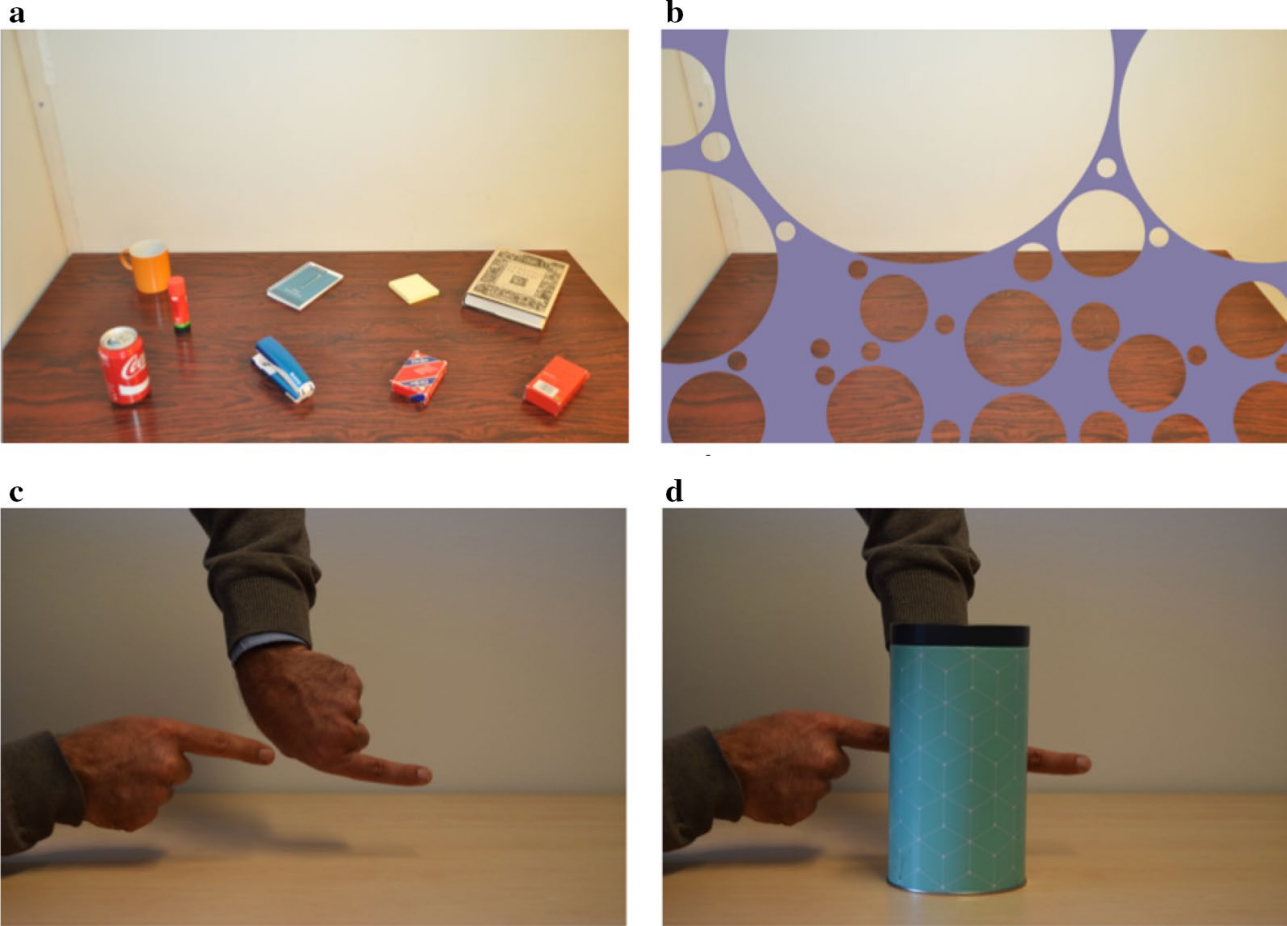
Top panels: A demonstration of the illusion of absence. Although all the objects in panel (**a**) are hidden behind the violet ”bubbled” occluder in panel **(b**), it is curiously difficult to imagine that they are really there. Bottom panels: A demonstration of amodal completion. The two fingers are experienced as a single long finger when they are partially occluded by the box (panel **d**). Note that this illusory impression persists even though it is quite absurd and contradicts your conscious knowledge. Top row adapted from The Other Side of Magic: The Psychology of Perceiving Hidden Things by V. Ekroll, B. Sayim and J. Wagemans, 2017, Perspectives on Psychological Science, 12(1), p. 98. Copyright (2017) by SAGE Publications. Reprinted with permission. Bottom row adapted from “Never repeat the same trick twice—unless it is cognitively impenetrable” by V. Ekroll, E. De Bruyckere, L. Vanwezemael and J. Wagemans, (2018), i-Perception, 9(6), p. 3, used under CC BY

The top panels in Fig. 1 show a static demonstration of the illusion of absence. All the objects on the table visible in panel (a) are hidden behind a violet “bubbled” occluder in panel (b), but notice how difficult it is to imagine that they are really there. Obviously, there is no direct visual evidence for or against the objects hidden behind the occluder, but we nevertheless experience an illusion which is reminiscent of the well-known cognitive fallacy of taking absence of evidence as evidence of absence. Many magic tricks may owe much of their impressive deceptiveness to this “illusion of absence” (Ekroll et al. 2017; Svalebjørg et al., 2020): By moving objects out of the perceptually empty space created by the illusion of absence, magicians can create the illusion that they appeared “out of nowhere”.^1^ Movie 1 in Øhrn et al. (2019, p. 3) shows a simple example, where the magician makes a coin apparently appear out of nowhere by pulling it out from the perceptually empty space behind his thumb. Richard Wiseman’s YouTube videos “The Mystery of the Red Cards”^2^ and “The Ball”^3^ show some further relevant examples. The Youtube video “Why This British Crossroads Is So Dangerous”^4^ contains a “virtual reality” simulation of a bicyclist suddenly appearing right in front of a car from the blind zone behind the roof support next to the windscreen (the so-called *A-pillar*). Note how surprising the sudden appearance of the bicyclist is, and how the experience of the event is very similar to the experience of many magic tricks.

### Can visual mechanisms determine our experience of invisible scene regions?

The above informal demonstrations are intriguing, but one may still be reluctant to accept the hypothesis that the magical experiences of appearances and disappearances and the corresponding impressions of absence are due to visual mechanisms. A priori, it does indeed appear rather counter-intuitive to suggest the visual mechanism determine our experience of occluded “blind spots” in the world since whatever is hidden in them obviously does not produce any visual stimulation at all. A large body of research on the well-known phenomenon of amodal completion, however, strongly suggest that this is indeed the case (Ekroll et al., 2018a, b; Ekroll et al., 2016; Gerbino, 2020; Kanizsa, 1985; Michotte et al., 1964; Scherzer & Faul, 2019; Scherzer & Ekroll, 2009, 2012, 2015; Shimojo & Nakayama, 1990; Van Lier & Gerbino, 2015). The bottom panels in Fig. 1 show an example of amodal completion. Note how the two aligned fingers are immediately and compellingly experienced as a single long finger when the “gap” between them is occluded behind the cylindrical can (panel d). The commonly accepted explanation for this kind of effect is that visual mechanisms create perceptual representations of hidden scene regions by performing various kinds of extrapolation^5^ based on the visible fragments of partially occluded objects (Thielen et al., 2019; Van Lier & Gerbino, 2015). The illusion of absence (top panels in Fig. 1) is similar to amodal completion (bottom panels in Fig. 1) in the sense that both phenomena involve strangely compelling impressions about what may or may not lie hidden behind occluding objects in the foreground, but there are differences. First, while amodal completion typically involve a curious sense of *presence* (Michotte et al., 1964), the illusion of absence consists in a curious sense of *absence* (Ekroll et al., 2017). Second, while amodal completion can be explained by appealing to various types of perceptual extrapolation of visible fragments, the illusion of absence does not involve any visible fragments that can form the basis of extrapolation. Hence, despite the phenomenological similarity between the two phenomena, they may be due to distinct underlying mechanisms. Therefore, the research supporting the conclusion that amodal completion is based on visual mechanisms does not necessarily imply that the illusion of absence is also based on visual mechanisms, although it does make the hypothesis more plausible. In the next section, we will briefly summarize recent evidence supporting this hypothesis.

### Direct experimental evidence suggesting that the illusion of absence is due to perceptual mechanisms

Since the illusion of absence was only recently described (Ekroll et al., 2017), it has thus far only been experimentally investigated in two studies (Øhrn et al., 2019; Svalebjørg et al., 2020). Both of these studies were designed to test the hypothesis that the illusion of absence is driven by perceptual mechanisms. A general feature of perceptual mechanisms is that they are cognitively impenetrable (Firestone & Scholl, 2016; Leslie, 1988; Pylyshyn, 1999). That is, the experiences they produce are not influenced by conscious knowledge, reasoning or expectations (even when the conscious knowledge directly contradicts the experience). A further tell-tale sign of perceptual mechanisms is that they (different from conscious knowledge or reasoning) have functional consequences within the perceptual system (Kanizsa, 1985; Scherzer & Ekroll, 2009, 2012; Shimojo & Nakayama, 1990). For instance, as Ekroll et al. (2016) have shown, an illusion of amodal volume completion (Gerbino & Zabai, 2003; Tse, 1999; van Lier, 1999; van Lier & Wagemans, 1999), where a semi-spherical shell viewed from the convex side is compellingly experienced as a complete ball, does not only persist when the shell is balanced on the viewer’s own finger, such that (s)he both feels and knows that it is really an empty shell, but also produces the illusion that the viewer's own finger has become shorter, as if to make space for the illusory volume of the “ball”. Øhrn et al. (2019) used a similar logic to investigate whether the illusion of absence can be attributed to visual mechanisms according to the criteria of (a) being cognitively impenetrable and (b) having functional consequences within the perceptual system. Their results show that a pencil resting on a vertical support is experienced as magically floating, if the support is occluded by a thin vertical strip. This happened even though the observers already had seen and thus knew about the existence of the support. It is difficult to explain that the observers experienced the pencil as magically floating despite their explicit knowledge that it was resting on the support without assuming that the occluder induced an illusion of empty space in the “blind spot” where the support was located. Thus, the results of this experiment strongly suggest that the illusion of absence is due to perceptual mechanisms. Svalebjørg et al. (2020) asked observers to view different magic tricks based on (a) various forms of attentional and reasoning misdirection, (b) amodal completion and (c) the illusion of absence. The task of the observers was to guess the secret behind the tricks, and each trick was presented three times. If a trick is based on a cognitively impenetrable perceptual illusion, one would predict that it should be very difficult to debunk, even after repeated presentations. The results confirmed this prediction for the tricks based on amodal completion and the tricks based on the illusion of absence, but not for the tricks based on attentional and/or reasoning misdirection. Thus, the results of this experiment also suggest that the illusion of absence is due to perceptual mechanisms.

### Preliminary theoretical explanation of the illusion of absence

**Fig. 2:**
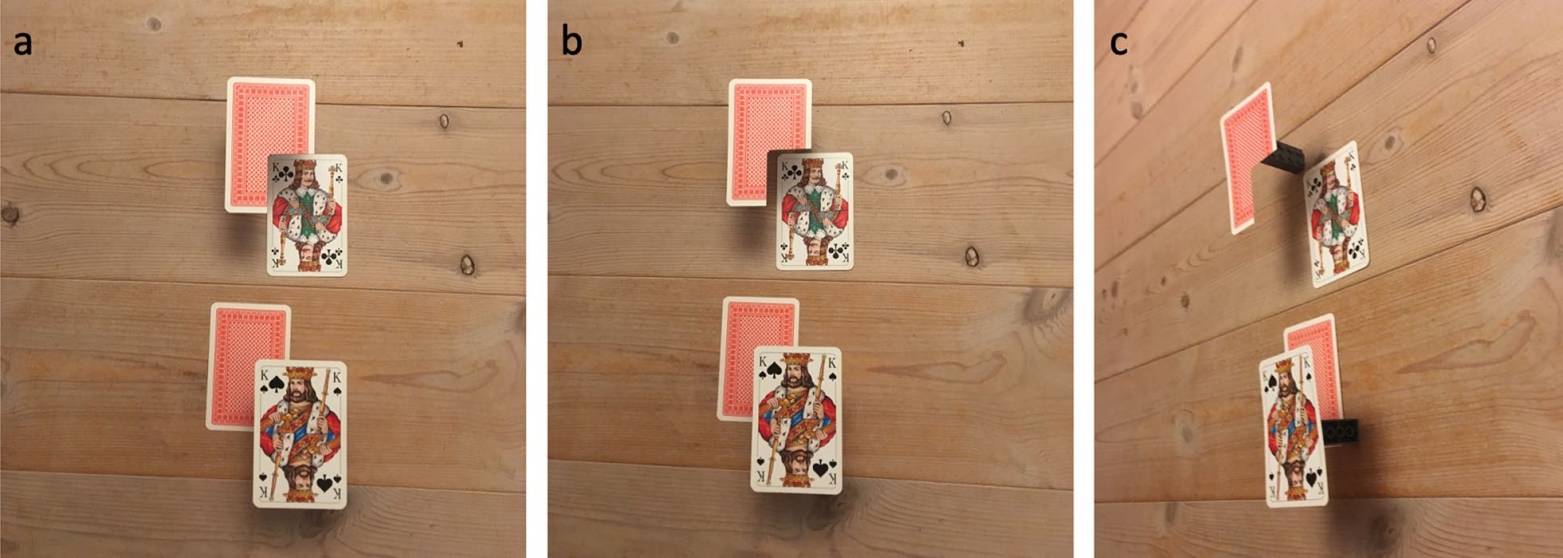
An example of a visual illusion that can be explained based on the principle of generic views. The three panels show the same four cards photographed from three different viewpoints. The picture card is in the front in the bottom row, and in the back in the top row, but in panel (**a**), it appears to be in front also in the top row. Adapted from Adelbert Ames’ “overlay demonstration” (Ittelson, 1952)

It is currently not established what perceptual mechanisms and principles underlie the illusion of absence, but it appears plausible to speculate that the mechanisms operate according to the generic view principle (Albert, 2001; Albert & Hoffman, 2000; Freeman, 1994; Koenderink & van Doorn, 1986; Nakayama & Shimojo, 1992). According to the generic views principle, the visual system assumes that the structure in the retinal image is qualitatively stable with respect to small changes in viewpoint. Figure 2 illustrates the generic view principle and its consequences using Adelbert Ames’ overlay demonstration (Ittelson, 1952) as an example. The three panels show the same spatial arrangement of four cards photographed from three different viewpoints. In the bottom row, the picture card is in the front but in the top row, it is in the back (although it appears to be in the front). The difference in viewpoint between panels (a) and (b) is small, but the structure in the corresponding retinal image changes qualitatively for the upper row, while it remains qualitatively the same for the bottom row. The illusory impression that the picture card is in the front in the top part of panel (a) can be attributed to the generic view principle: Because the interpretation that the picture card is in the front is compatible with the assumption that the retinal image is qualitatively stable with respect to small changes in viewpoint, the visual system prefers this interpretation over the correct one.

The generic view principle can be understood as a heuristic that aids the visual system in inferring the most likely interpretation of the ambiguous retinal input by excluding interpretations that would involve highly unlikely coincidences (Rock, 1983; Van Lier et al., 1994). This is because the retinal image of a visual scene is qualitatively stable with respect to small changes in viewpoint in the vast majority of cases, and qualitatively unstable only in very rare cases such as the one on top of Fig. 2a (see, e.g. Koenderink & van Doorn, 1986).

The generic view principle readily explains why the broomstick in Movie 2 in Øhrn et al. (2019, p. 12) is experienced as a ball rather than as the stick it actually is. It also readily explains why the space behind the illusory ball is experienced as empty. Actually, all the demonstrations of the illusion of absence we have described above are readily explained by appealing to this principle. Magic tricks relying on the illusion of absence (Svalebjørg et al., 2020) involve a very special alignment of the occluder and the hidden object along the line of sight. If the tricks were viewed from a somewhat different viewing position, the hidden object would have been visible, and the trick ruined. This is why magicians take care to “watch their angles” (e.g. Bobo, 1982; Macknik et al., 2010). Similarly, creating the static demonstration of the illusion of absence in Fig. 1 required careful alignment of the bubbled occluder so that it would cover all the objects on the table. A straightforward prediction of the hypothesis that the illusion of absence is due to mechanisms operating according to the principle of generic views is that the illusion of absence should be more likely to occur or be stronger for a very small (or narrow) occluder than a bigger (or broader) one. The reason for this is that if a narrow occluder is to be occluding another object, however small, the occluder and the occluded object must be very narrowly aligned along the line of sight (except when the occluded object is very close to the occluder). A broader occluder, on the other hand, allows for many more possible positions of the occluded object relative to the occluder. Øhrn et al. (2019) tested this prediction using both a narrow occluder and a wider one. As predicted by the generic view principle, the illusion of absence (as measured indirectly via the floating illusion) was weaker for the broader occluder.

Although the results of Øhrn et al.’s (2019) study supports the hypothesis that the principle of generic views underlies the illusion of absence, further testing of the theory is necessary before strong conclusions can be drawn, and alternative candidate explanations should be developed and tested. One alternative explanation could be that the perceptual system is biased against more than one object representation at the same location of the visual field, such that the representation of the occluder vetoes the possibility of other perceptual objects behind the occluder, in loose analogy with the phenomenon of object substitution (Enns & Di Lollo, 1997).

## Potential involvement of the illusion of absence in road accidents

The above demonstrations and findings strongly suggest that visual mechanism may indeed, in some cases, evoke a powerful illusion that the space behind an occluding object in the foreground is empty. If this illusion occurs in traffic situations, it could obviously pose a serious hazard, because the illusion would create a misleading confidence that the road behind an occluding object in or outside of the car is free. In principle, the illusion of absence may be implicated in all accidents involving obstructions of view due to occlusion, but this seems implausible. Obviously, not every case of occlusion evokes the illusion that the space behind the occluder is empty. In many cases, particularly when the occluding object is large in our field of view, we are acutely aware of the fact that we cannot know what may be hidden behind it. For example, short sight distances at intersections and roundabout are often associated with reduced speeds (Angelastro, 2011; Schepers et al., 2011). In line with the above hypothesis that the illusion of absence is based on the principle of generic views, we assume that the illusion of absence is most pronounced in cases where the occluding object is relatively small or narrow in the field of view, but more research is needed to establish how the strength of the illusion depends on occluder width as well as other parameters that are likely to be important, such as the motion/trajectory of the occluder and the length of time it is perceived without revealing any objects coming out from behind it. This will require more systematic parametric research which is beyond the scope of the present paper. We shall now consider some illustrative traffic scenarios where the illusion of absence may be a risk factor.

### A-pillar obstruction

**Fig. 3:**
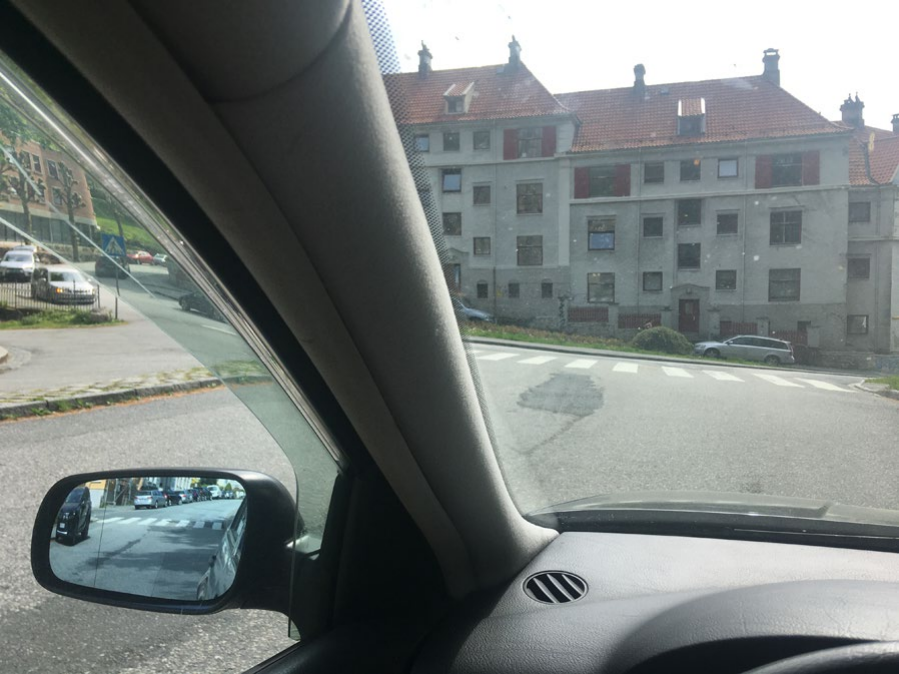
The A-pillars next to the windscreen can hide the view of other road users

**Fig. 4:**
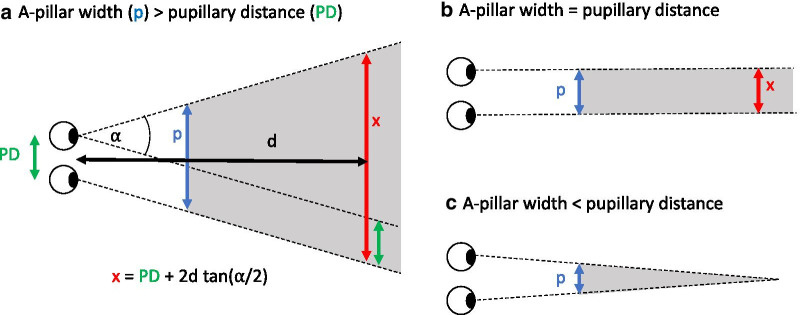
Schematic illustration of the binocular blind zone (gray regions) created by A-pillars (blue arrows) of different widths. **a** If the A-pillar is wider than the distance between the pupillary distance PD—which is the case in most extant cars—, the width (x) of the blind zone *increases* with the distance (d) from the observer. **b** If the width of the A-pillar were equal to the pupillary distance, the width of the binocular blind zone would be equal to the pupillary distance (typically about 6 cm) at any distance. **c** If the width of the A-pillar were less than the pupillary distance, the width of the blind zone would *decrease* with distance. Panel (**a**) also illustrates the definition of the binocular obstruction angle α. The equation underneath shows how the width *x* of the binocular blind zone at a given distance *d* can be calculated based on the binocular obstruction angle and the interpupillary distance. In (**b**), the binocular obstruction angle is zero, and in (c) it is negative

As is well known, the A-pillars (see Fig. 3) located on the sides of the windshield in cars and other vehicles create forward-looking blind spots which can easily hide pedestrians, bicyclists or motorcyclists, even at comparatively short distances from the car (Beach, 2004; Green, 2018; Marshall et al., 2012; Millington et al., 2006; Quigley et al., 2001; Reed, 2008; Remlinger, 2013; Road Research Laboratory, 1963; Vargas-Martin & Garcia-Perez, 2005; Wade & Hammond, 2002). The region which is invisible to both eyes depends on the A-pillar design, the position of the driver relative to the A-pillar and the distance between the driver’s two eyes (interpupillary distance). As illustrated in Fig. 4, the width of the binocular blind zone increases with distance when the A-pillar is wider than the interpupillary distance (Fig. 4a), but remains constant when the width of the A-pillar equals the interpupillary distance (Fig. 4b) and decreases with distance when the width of the A-pillar is less than the interpupillary distance (Fig. 4c). Thus, for drivers with vision in both eyes, A-pillars of a width equal to or less than the interpupillary distance are not likely to pose any significant hazard because any object wider than the interpupillary distance, which is typically about 6 cm, would be visible to at least one eye at any distance. The A-pillars are however normally wider than that, particularly in modern cars (Quigley et al., 2001), such that the width of the A-pillar blind zone increases with distance. Measurements with a sample of different cars indicate an average binocular obscuration angle (see Fig. 4a) of 7.3° (range 5.4° to 9.4°) for the A-pillar on the driver's side (Quigley et al., 2001, their table 9). Thus, with the average binocular obscuration angle (7.3°), a pedestrian which is 0.4 m wide can be totally occluded at all distances larger than 2.7 m, a bicycle which is 1.7 m long at all distances larger than 12.9 m and a car which is 4.5 m long at all distances larger than 34.8 m. In actual scenarios, the other road users may actually be occluded at even shorter distances, for two reasons: First, Quigley et al.’s (2001) measurements of the obscuration angles were based on a driver seated in the most rearward position possible, but drivers are often seated further towards the front, and hence closer to the A-pillar, where the obscuration angle will tend to be larger. Second, the other road users will often be oriented obliquely relative to the line of sight, such that they effectively project a smaller width orthogonal to the blind zone. Many different situations have been described where A-pillar obstruction may pose a serious hazard (Beach, 2004; Bez, 2018; Millington et al., 2006; Road Research Laboratory, 1963). We shall not attempt to give an exhaustive overview of the many relevant collision scenarios, but rather focus on a few simple cases, which appear particularly revealing and well-suited for clarifying the potential role of the illusion of absence.

### Lateral collisions with other road users while driving straight ahead

**Fig. 5:**
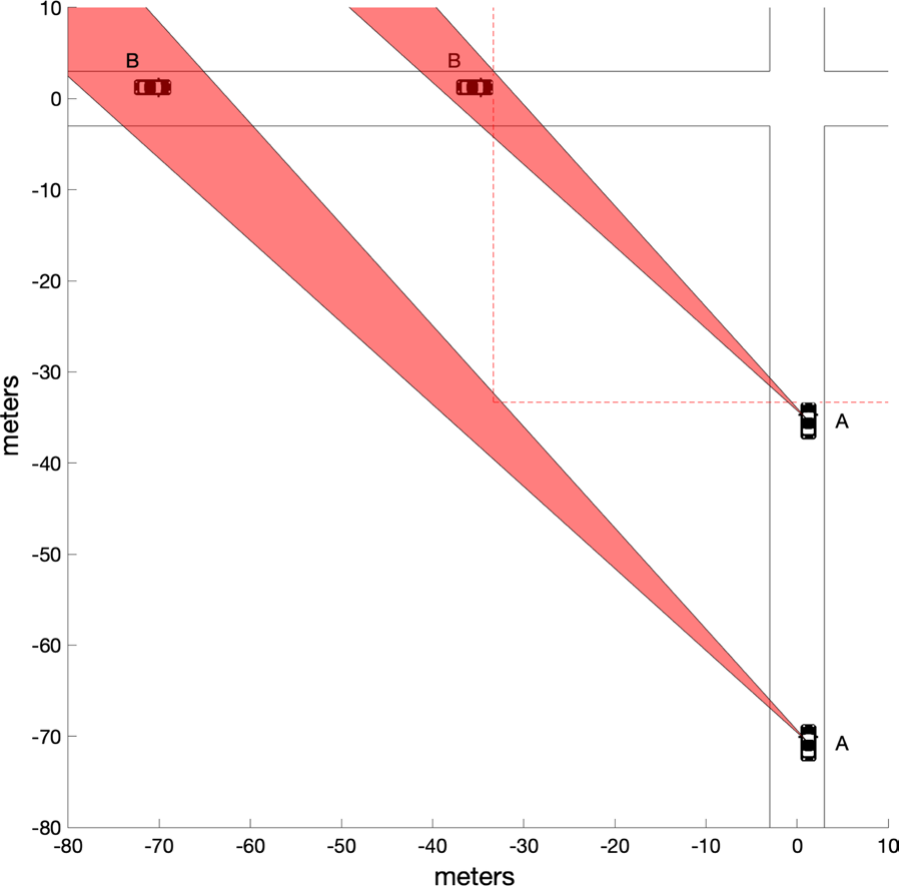
Illustration of how car B may remain trapped in the A-pillar blind zone (red region) of car A until after they have passed a conservative estimate (reaction time 1.5 s., dry road) of the stopping distance for cars riding at 50 km/h (red dotted lines). The cars are shown at two different points of time. In this example, equal speeds are assumed. The A-pillar bearing is 45° and the A-pillar obstruction angle is 7.3°, which are realistic average values (see text). Note that the A-pillars of some vehicles have considerably larger obscuration angles, and that the obscuration angle may increase even further if the driver is sitting closer than the most rearward position of the seat. The cars are 4 m long and 1.75 m wide, which corresponds to the measures of a typical four-person car (e.g. VW Polo)

**Fig. 6:**
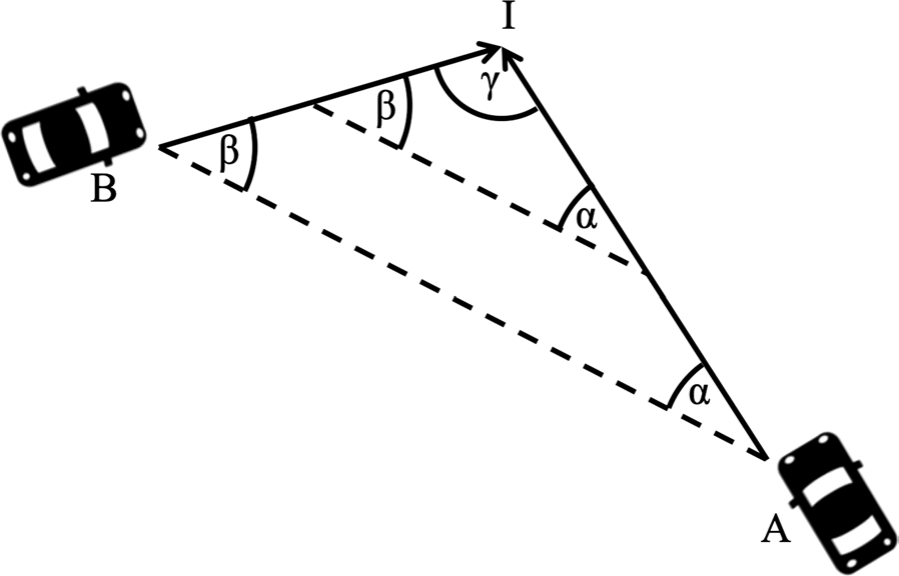
Illustration of the CBDR principle. Two vehicles A and B are driving on straight paths that intersect at the point I, each with their own constant speed. If the bearing α of vehicle B viewed from vehicle A remains constant at all times, the vehicles are going to reach the intersection I simultaneously, i.e. they are going to collide. Note that, by symmetry, this also means that the bearing β of vehicle A is constant. If the bearing α decreases with time, vehicle B will have passed the intersection when vehicle reaches it (this is only true in a strict sense if we neglect the size of the vehicles). Conversely, if α increases, vehicle B will not yet have reached the intersection. Note that these rules are valid irrespective of the angle γ between the straight paths

Figure 5 illustrates a scenario where a car (B) approaching from the left is trapped in the blind zone (red area) created by the A-pillar of another car (A) driving straight ahead. The cars are shown at two different points of time, and B can remain completely trapped in the blind spot until after both cars have passed a conservative estimate of the stopping distance for a speed of 50 km/h (red dotted lines). There are many different ways in which the speeds and accelerations/decelerations of the vehicles may co-vary such that B remains trapped in the blind zone of A until just before impact. Since the intersection of the blind zone with the path of the B is larger at earlier points of time, there is additional leverage for imperfect co-variation in speed. In the special (but not unlikely) case where both road users are travelling at constant speeds, it is straightforward to work out what speed ratios will lead to total A-pillar obstruction of B until right before impact because a simple geometrical rule known as the constant-bearing-decreasing-range (CBDR) principle (Bez, 2018; Cutting et al., 1995; Green, 2018; Lenoir et al., 1999; Morris, 2005; Remlinger, 2013) can be applied. According to this principle (Fig. 6), the bearing α of road user B viewed from road user A stays constant if the speed ratio is such that the two vehicles are going to collide. Furthermore, for any arbitrary angle γ between the paths of the two vehicles, this speed ratio is uniquely related to the bearing angle α. Given a right-angle intersection, the bearing angle α = 45° corresponds to a speed ratio of unity, meaning that two road users must drive at the same speed. A ballpark estimate of the typical bearing of the A-pillar on the driver's side (based on measurements reported in Quigley et al., 2001) is 46°, meaning that if the two road users move at roughly equal speeds, road user B may remain trapped in the centre of road user A's A-pillar blind zone until right before impact. Importantly, there is not just a single speed for which road user B may remain trapped in the blind zone. Rather than being trapped in the centre, its back end may track the back end of the blind zone, or its front end may track the front end of the blind zone. Consequently, in the situation at hand, and assuming an A-pillar obstruction angle of 7.3° (the average value reported by Quigley et al., 2001), it can be calculated that a range of different speed ratios varying with a factor of about 1.3 are all compatible with total A-pillar obstruction until right before impact. Note that the obscuration angle is larger than 7.3° for some vehicles (Quigley et al., 2001). Furthermore, Quigley et al.’s (2001) estimates of obstruction angles were made on the assumption that the driver was seated in the most rearward position possible, meaning that the obstruction angles can be considerably larger for drivers seated closer to the front (and hence closer to the A-pillar). Thus, although continual occlusion of a second road user behind the A-pillar requires quite a large amount of coincidence, it does not require a perfect coincidence. And even though it is not very likely to happen as such, the absolute number of such coincidences may be considerable given the large amount of traffic in modern society.

It is also worth noting that by virtue of being located at a constant bearing relative to the direction of travel, the A-pillar creates a forward-looking blind spot that is more dangerous than most other blind spots that occur in natural locomotion. Since constant bearing means that collision is likely, while changing bearing means that it is less likely, the A-pillar can be said to create the most adverse visibility conditions in the most dangerous situations! Note that this unfortunate regularity or “coincidence” is a consequence of having an obstruction of view positioned at constant bearing relative to the direction of travel from the observer’s point of view. Thus, the unfortunate regularity is characteristic of obstructions of view being part of (or attached to) a vehicle, but not of other obstructions of view, such as those outside of the vehicle or the obstructions of view typically encountered by an observer who does not travel in a vehicle (e.g. a pedestrian).

As already mentioned, it appears plausible that the A-pillar will provoke the illusion of absence since it is relatively narrow. Based on the principle of generic views, one would also expect that the tendency to experience the illusion of absence is particularly strong for moving occluders such as the A-pillar, because prolonged total occlusion of an object behind a moving occluder requires a higher degree of coincidence than prolonged total occlusion in static situations such as the one studied by Øhrn et al. (2019). Some observations made by Wade and Hammond (2002) in a virtual reality experiment investigating the present kind of traffic scenario suggest that an illusion of absence is indeed evoked by the A-pillar. For instance, they noted that “participants sometimes expressed mild anger at being *tricked or fooled* into a collision” (ibid, p. 17, our emphasis) and that the “comment was often made that the car just “appeared” at the intersection” (ibid, p. 18). The illusion of absence may have several important consequences in this and similar scenarios:

First, it may play an important role in creating a misleading sense of security, and thus prevent the driver from taking appropriate precautions, such as checking whether the blind spot is empty by moving the head back and forth (Habib, 1980; Marshall et al., 2012; Wade & Hammond, 2002). Indeed, the findings of Wade and Hammond (2002) suggest that drivers do not engage in such active scanning very often. Similarly, Remlinger (2013), who performed a similar virtual reality experiment, concluded that “only some of the participants exhibited pronounced search- or avoidance movements. Instead, it appears that the human perceptual processing of many drivers cover up the missing parts of the visual information. Movements aiming at the detection of potentially dangerous occluded objects are therefore frequently not initiated” (p. 216, our translation of the original German).

Second, the illusion of absence may play an important role in preventing road user A from noticing road user B even when the latter is only hidden in the A-pillar blind zone for a brief period of time. As is well known, visual resolution is rather limited in the retinal periphery, such that gaining a good overview of the larger scene in front of you requires a series of changes in fixation (saccades, Hardiess et al., 2013; Land, 2006). Furthermore, as is also well known, even if we look straight at something, we might fail to see it due to inattentional blindness (Kuhn & Tatler, 2011; Mack & Rock, 1998; Macknik et al., 2008; O’Regan et al., 2000; Simons & Chabris, 1999). Thus, visual information is to a considerable extent sampled from the visual scene in a serial fashion (Noton & Stark, 1971), where the parts that are sampled (in terms of overt and covert attention) are determined not only by low-level saliency, but also by expectations or mental plots (Itti & Koch, 2000; Koenderink, 2019; Rensink et al., 1997). Thus, it appears plausible that a driver riding straight ahead may pay most attention straight ahead, while taking only a brief look to the side to check for crossing traffic when approaching an intersection. If a crossing road user is hidden in the blind zone of the A-pillar at that moment, and the driver experiences the illusion of absence, the driver may already be convinced that the road is free and therefore fail to take a second look.

Third, if a driver fails to check for other road users hidden behind the A-pillar and an accident occurs, it may otherwise appear reasonable to charge her or him with negligence, but if the reason for the failure to check is a powerful visual illusion, which is regularly used by magicians to create illusions of impossibility, the driver can hardly be reasonably regarded as negligent.

Lastly, when evaluating the potential merits of different conceivable countermeasures aiming at reducing the risk of accidents associated with A-pillar obstruction, interventions based on improvements in A-pillar (Pipkorn et al., 2012; Quigley et al., 2001; Vaidya et al., 2017) or road design (Bez, 2018) would appear more promising than attempts at influencing driver awareness or behaviour (Marshall et al., 2012) if the illusion of absence plays a central role. The argument for this is that if the illusion of absence is indeed a visual and cognitively impenetrable illusion it may be very difficult or even impossible for people to acquire strategies and behaviours that would reliably make them immune to its deceptive powers.

**Fig. 7:**
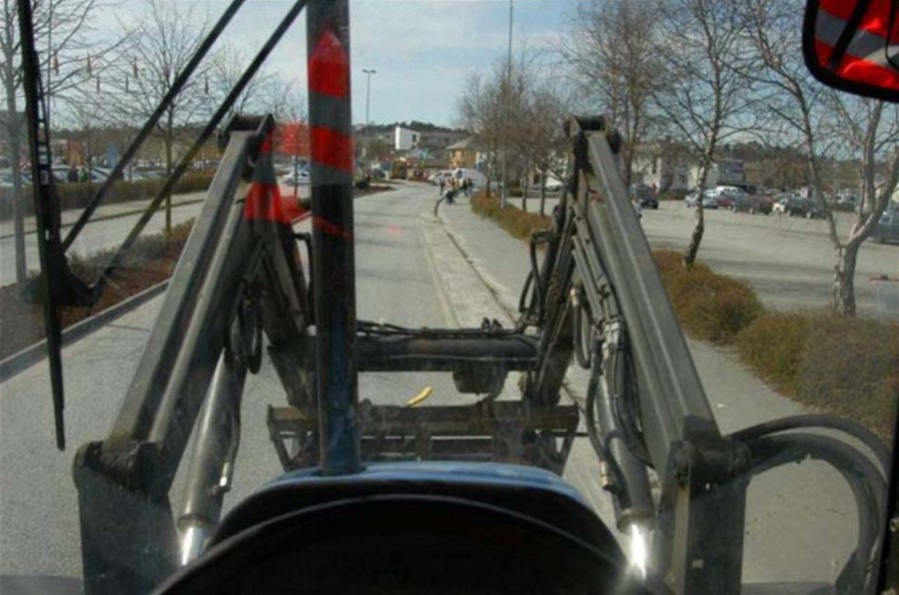
The front end loader on tractors or similar vehicles also creates forward-looking blind spots, which have similar basic properties as those created by A-pillars. From Ringen and Moss-Iversen (2017), reprinted with permission

In the above, we have focussed on forward-looking blind spots created by the A-pillars as an example, but it should be kept in mind that essentially the same line of reasoning applies to other forward-looking blind spots such those created by mirrors, navigation displays or the front end loader of a tractor (Ringen & Moss-Iversen, 2017; see Fig. 7).

### Turning vehicles hitting a pedestrian

When a car or bus driver makes a left or right turn at an intersection, pedestrians crossing at the crosswalk which runs parallel to the vehicle’s direction prior to the turn are at peril of getting trapped in the A-pillar blind spot (seeFig. 8).^6^ Some observations (Habib, 1980; Lord et al., 1998; National Academies of Sciences, Engineering, and Medicine, 2008) suggest that the risk is greater for left turns than for right turns in countries where the driver’s seat is located on the left, and conversely in countries where the driver’s seat is located on the right. In principle, several factors may account for this pattern (Habib, 1980; Lord et al., 1998), but it is notable that the A-pillar obstruction during the turning manoeuvre is greater for the A-pillar on the driver’s side (Abdulsatter & McCoy, 1999). Since the A-pillar on the driver’s side is closer to the driver, it occludes a larger visual angle. Furthermore, the blind spot created by the A-pillar on the driver’s side may sweep across the crosswalk twice during the turn. Before the turn it sweeps across the crosswalk in forward direction, and during the turn it sweeps across the crosswalk in the backward direction. As in the above case with lateral collision while driving straight ahead, it is also here possible that another road user (most plausibly a pedestrian, in this case) gets trapped in the A-pillar blind zone for an extended period of time and until right before impact. Also as in the above case, even if the other road user is only occluded for a short period of time, the illusion of absence may plausibly inhibit further visual scanning behaviour that would make the driver notice the pedestrian when (s)he is actually visible. Indeed, in the left-turn scenario, it may be even more likely that the driver directs her or his gaze towards the dangerous zone (the crosswalk) infrequently and briefly due to the many competing attentional demands (such as the need to check for traffic from straight ahead).

**Fig. 8:**
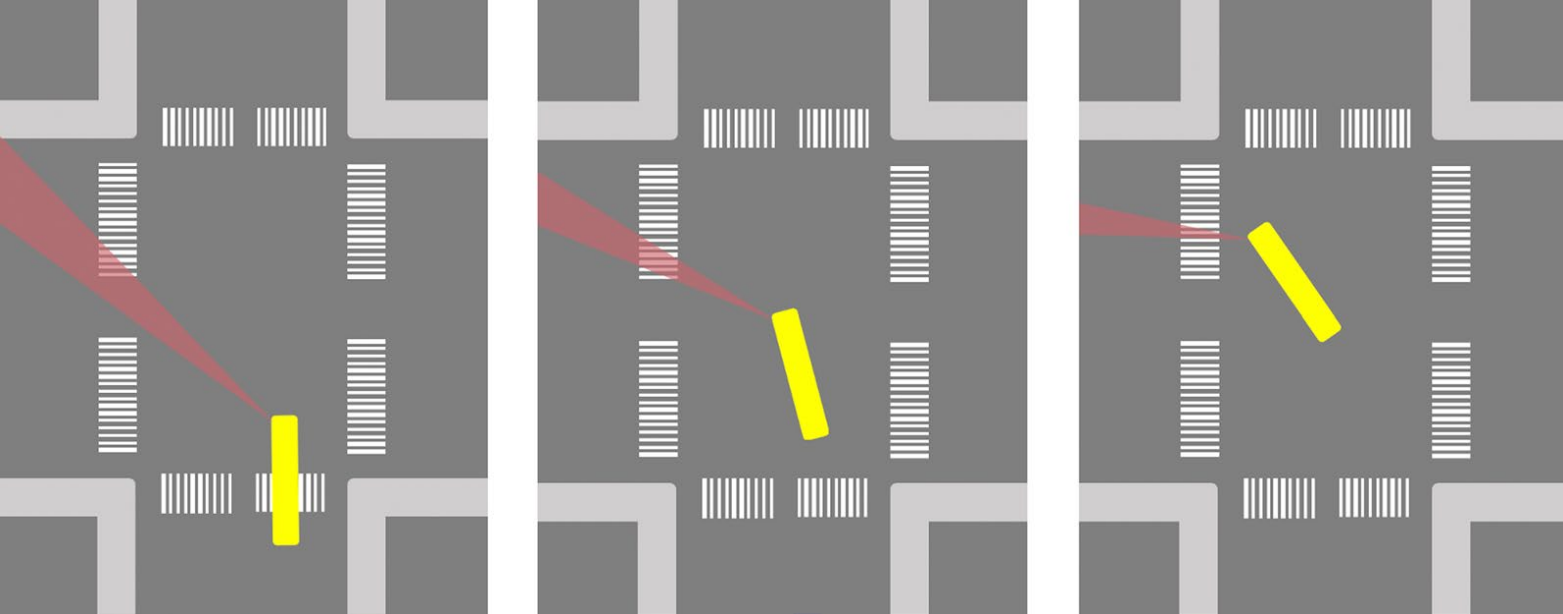
Schematic illustration of how the A-pillar blind zone (red area) of a left-turning bus (yellow) may sweep across a crosswalk and potentially obscure a crossing pedestrian until right before impact

### Obstructions of view positioned outside of the car

**Fig. 9:**
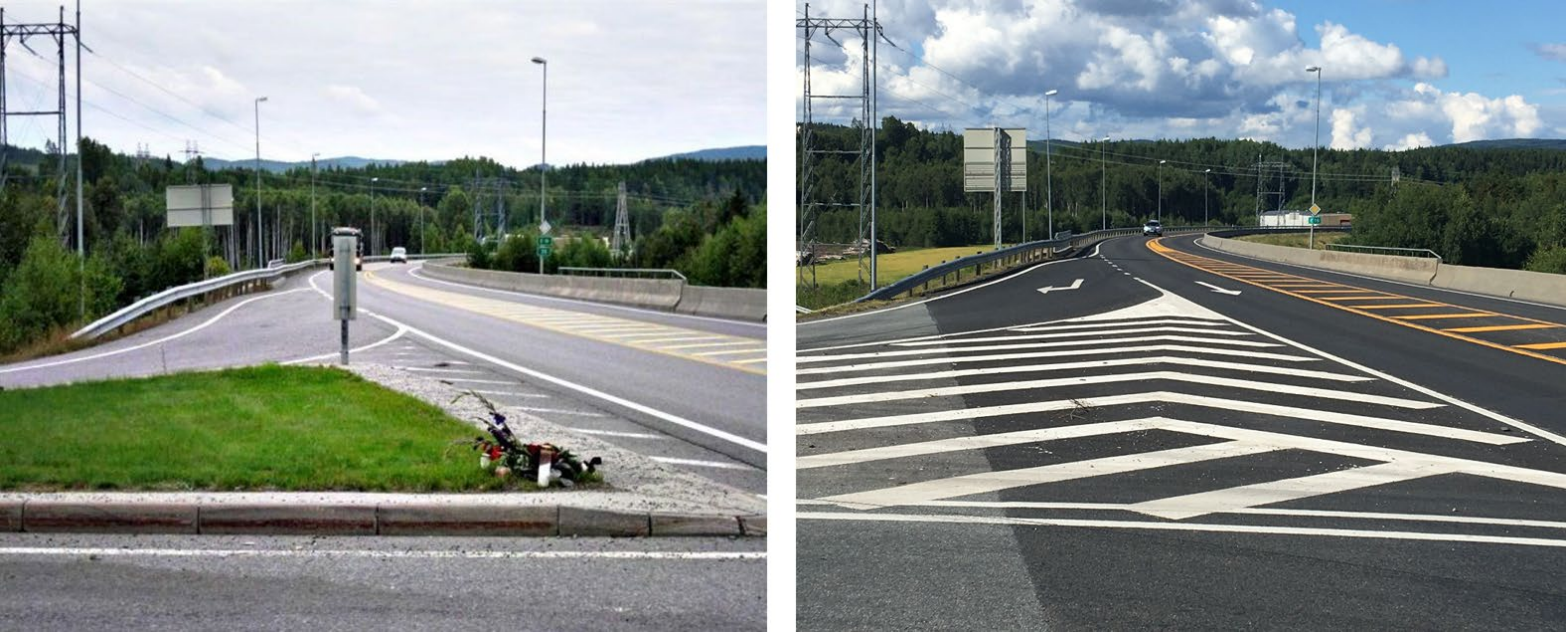
(Left) The narrow signpost in the middle can hide a motorcycle approaching the intersection over a long stretch (150 m, see Haakenstad, 2015) from the point of view of a driver halting at the entry to the main road. In this photo, a large truck is almost completely covered. This probably happened during a fatal accident at this intersection (Amundsen et al., 2015). Based on the findings of Øhrn et al. (2019), one may speculate that narrow obstructions of view like this signpost are particularly prone to evoking the illusion of absence, and thus a compelling, but potentially misleading conviction that it is safe to enter the main road. (Right) The signpost has later been removed. The photo on the left was taken by Pål Bjerke, Norwegian Public Roads Administration and is reprinted with permission

The above analysis suggests that the potential of the A-pillar or other in-vehicle obstructions of view for hiding other road users who are on a collision course is greater than one might intuitively expect. In contrast, the risks associated with obstructions of view positioned outside of the car are perhaps more obvious. If the risk is obvious to the driver, it appears reasonable to assume that (s)he will take appropriate precautions, like reducing speed and increase visual scanning of areas where another road user may suddenly pop out. When the object creating the obstruction of view is large, one may plausibly expect that the driver will be aware of the risk. Indeed, it has been argued that intentionally creating environmental obstructions of view could actually increase traffic safety because drivers approaching an obstructed intersection may drive more carefully (Green, 2018). In the case of smaller obstructions of view, however, road users may be less aware of the associated risks: First, a smaller obstruction is probably less likely to be noticed. Second, the illusion of absence, which creates a misleading sense of security, may be more likely to be evoked by smaller than by larger obstructions of view (Øhrn et al., 2019). On the other hand, a small, stationary obstruction of view cannot hide another road user for very long if the viewer is moving. If the viewer is stationary, however, and the other road user moves on a roughly straight path aligned with the blind spot, the risk can be greater. A fatal accident that occurred when a car entered a main road at intersection with a stop sign, colliding with an approaching motorcycle illustrates this point (Amundsen et al., 2015). Figure 9 (left) shows the driver’s view from the location where he was required to halt at a stop sign before entering the main road. In the photo, a small, vertically oriented chevron road sign can be seen to almost entirely obstruct the view of a large approaching truck. Importantly, this small road sign may hide a smaller road user like the approaching motorcycle for a long stretch (150 m, see Haakenstad, 2015) while it is riding straight ahead along the main road. This example illustrates that a rather small obstruction of view may obscure an approaching road user’s path for a long stretch until right before impact if the viewing driver is stationary, which s(he) is required to at an intersection regulated by a stop sign. In such a situation, it seems plausible that the illusion of absence can occur, particularly because the obstruction of view is small (Øhrn et al., 2019). Thus, the driver entering the main road may have the compelling impression that the road behind the road sign is free, and mistakenly believe that it is safe to enter the intersection.

## Discussion

It is obvious that obstructions of view pose a danger in traffic because they may hide other road users on collision course from view. Assuming that people are consciously aware of the actual lack of visibility produced by obstructions of view, conscientious and responsible drivers may be expected to exhibit appropriate caution and check for other road users potentially hidden in the blind zones behind the obstructions of view. In this article, however, we have delineated how the recently discovered illusion of absence (Ekroll et al. 2017; Øhrn et al., 2019; Svalebjørg et al., 2020) may limit peoples’ ability to experience the actual lack of visibility in some situations and therefore compel them to underestimate the potential for collision with other road users hidden in blind zones. We have pointed out that many informal demonstrations as well as the presently available experimental evidence (Øhrn et al., 2019; Svalebjørg et al., 2020) agree in suggesting that although the illusion of absence refers to invisible regions in a visual scene, it is nevertheless driven by visual mechanisms and that it can be just as deceptive and powerful as ordinary visual illusions and magic tricks.

We have also discussed concrete examples of real-life scenarios where this illusion of absence may heighten the risk of traffic accidents. These examples are illustrative and by no means exhaustive, and further work is needed to develop a more comprehensive and systematic overview of traffic scenarios where the illusion of absence may play a role.

In the section “Preliminary theoretical explanation of the illusion of absence", we suggested that the illusion of absence may be explained by appealing to the principle of generic views (Albert, 2001). According to this hypothesis, the illusion should be more powerful and/or likely to occur with small or narrow obstructions of view. Experimental work provides some evidence for this hypothesis (Øhrn et al., 2019). This hypothesis implies that the relationship between the size of an obstruction of view and its potential for contributing to serious traffic accidents may be less straightforward than one may intuitively assume. Although larger obstructions of view are generally more likely to obstruct the view of other road users, smaller obstruction of view may be disproportionally dangerous because they are more prone to generating a misleading sense of security.

Several different kinds of research are needed in order to assess to what extent and under which conditions the illusion of absence contributes to traffic accidents and to develop appropriate countermeasures. At the most fundamental level, we need to know more about the general stimulus factors that evoke the illusion of absence and to develop a general model of the underlying perceptual principles. At present, there is some evidence suggesting that the illusion of absence is more likely to occur for small obstructions of view than for larger ones, in line with the idea that the illusion of absence is based on the perceptual principle of generic views (Øhrn et al., 2019), but more research is needed to gain firmer and more general knowledge of the relevant stimulus factors. The study of Øhrn et al. (2019) investigated the illusion of absence in a static situation, but it appears plausible that the illusion may be even more pronounced in dynamic situations, and this needs to be investigated in future experiments. Based on insights from basic research addressing these questions, we envision that it should be possible to predict in what typical traffic scenarios the illusion of absence is most likely to pose a significant risk.

But even when the critical stimulus conditions for the illusion of absence are known, significant research efforts are needed to predict its impact in real-life traffic scenarios. By way of example, consider the scenario described in the section “Lateral collisions with other road users while driving straight ahead” and Fig. 5. If another road user remains completely trapped in the A-pillar until right before impact, this is clearly a dangerous situation, that may be even more dangerous due to the illusion of absence, but assessing how frequently such accidental alignments of vehicle paths can be expected to occur in real-world scenarios is non-trivial. As we have argued, the probability that this occurs is non-zero, but more sophisticated analyses like traffic flow simulations (microsimulations) using plausible assumptions about parameters such as the natural distributions of vehicle speeds, starting positions and density of traffic are necessary to assess exactly how frequently this is to likely to occur. It is also necessary to investigate how the illusion of absence may interact with other cognitive factors such as eye scanning behaviour, attention and cognitive load in real-life situations: As explained in the section “Lateral collisions with other road users while driving straight ahead”, the illusion of absence occurring in a single glance may plausibly inhibit further visual scanning and allocation of visual attention, and thus possibly pose a risk even when the other road user is occluded only for a brief period of time. To investigate this possibility, experimental investigations using virtual reality simulations of corresponding traffic scenario could be revealing. It would of course also be of interest to analyse to what extent there is evidence for a contribution of the illusion of absence in existing accident reports and statistics. Such an endeavour is difficult, however, considering that relevant information may not have been collected in on-the-spot investigations. We hope, however, that our analysis may encourage on-the-spot accident investigators to consider the possible involvement of the illusion of absence and to collect relevant data that can be analysed in the future.

If future research confirms that the illusion of absence indeed contributes to traffic accidents, several important conclusions can be drawn. While failing to check for other road users that may be hidden behind an occluder may be considered negligent, it is more difficult to see how a driver may be expected to anticipate and take precautions against a hitherto unknown and deceptive visual illusion, which is powerful enough to even create magical experiences. Scientific knowledge about the illusion of absence can have implications for the plausibility of statements of defendants claiming to have taken precautions to ascertain that the road was free, but that another road user nevertheless seemed to “appear out of nowhere”. Considering that the illusion of absence is a subjective visual illusion that is triggered by specific visual input, it can be hard to appreciate for investigators and parties in court proceedings unless they experience it themselves. Thus, VR simulations/reconstructions of the accident from the driver’s point of view may furnish a particularly useful tool for fair assessment.

More research on and better scientific knowledge about the illusion of absence may also speak to the effectiveness of different legal practices with respect to the aim of reducing the risk of accidents. As discussed by Remlinger (2013), primary responsibility for accidents where A-pillar blind zones are implicated are routinely and exclusively assigned to the driver in the German legal system. In a ruling from a higher regional court (Oberlandesgericht Hamm, dated 31.08.2000) for instance, it is concluded that the A-pillar blind zone is not an exonerating factor “because the driver can neutralize it without any problems by changing the position of the head” (our translation from the German excerpt cited in Remlinger, 2013, pp. 63–64). If future research confirms that the illusion of absence plays a significant role, such that drivers are often oblivious to the need for such neutralization, routinely assigning responsibility to driver may be less effective in reducing the risk accidents than pursuing improvements in A-pillar design. A related, potentially problematic consequence of the German legal practice is that it may limit the possibilities for gaining a realistic estimate of the prevalence of accidents related to A-pillar obstruction. According to Remlinger (2013), the concept “obstruction of view” is only used for obstructions of view located outside of the vehicle in German legal practice, while obstructions of view belonging to an accredited vehicle (such as the A-pillar) are regarded as non-existent or irrelevant from a legal point of view. As a consequence, they are not considered as obstructions of view in police reports and legal proceedings. Furthermore, statements from parties involved in the accidents regarding such obstructions of view are often not registered because they would entail self-incrimination (Remlinger, 2013).

Improved scientific knowledge of the potential role of the illusion of absence in traffic accidents would also have implications for the development and evaluation of preventive countermeasures. With regard to the specific risks posed by A-pillar obscuration, for instance, both driver-based educational approaches and environment-oriented approaches targeting the vehicle and/or road design are conceivable, but if a cognitively impenetrable illusion like the illusion of absence is involved, the former may be less effective. With respect to driver-based approaches like encouraging longer gaze durations, Crundall et al. (2008, pp. 19–20) note that.

“One problem with this approach is that drivers may theoretically understand the potential for windscreen pillars to obscure the road, yet may fail to heed the advice when it is needed. This is because the situation does not necessarily provide clues to the problem. The windscreen pillar may act in a similar fashion to the retinal blind spot.”

Similarly, Remlinger (2013, p. 38) have suggested that the area behind the A-pillar may be perceptually filled in based on the Gestalt principles of closure and good continuation. Both of these suggestions are very much in line with our suggestion that the A-pillar evokes an illusion of absence driven by visual mechanisms, although we go further in positing that visual mechanisms not only fill in the invisible parts of the background from context, but also exclude the possibility that anything is located in the 3D space *between* the perceptually filled-in parts of the background and the occluder. Thus, in our analysis, drivers “may fail to heed the advice when it is needed” not only “because the situation does not necessarily provide clues to the problem”, but because the visual system creates the compelling visual illusion that there *cannot be a problem.* Road safety campaigns informing drivers about the dangers of A-pillar view obstructions and the illusion of absence may provide drivers with conceptual knowledge that may ameliorate the problem to some extent, but two considerations suggest that their effectiveness need to be evaluated carefully. First, a meta-analysis suggests that road safety campaigns that are not accompanied by enforcement have little or no effect on crashes (Elvik et al., 2009; see also Hoekstra, & Wegman, 2011). Second, given that the illusion of absence is a cognitively impenetrable illusion that persists in the face of better knowledge, it may be very difficult for drivers to overcome their natural and automatic tendency to trust “what they see with their own eyes” and apply conceptual knowledge that would contradict their immediate perceptual experience, particularly in natural driving situations which also require attention to other, directly visible road users.

In light of this, environment-oriented approaches targeting the vehicle and/or road design may be a necessary complement to road safety campaigns and driver training. If future research shows that the risk due to A-pillar obstruction is indeed larger than hitherto believed due to the illusion of absence, car manufacturers may have reason to invest more resources into the development and implementation of car-design solutions that would reduce or eliminate A-pillar bind zones. Solutions that have been proposed include reducing the A-pillar width (Pipkorn et al., 2012; Vaidya et al., 2017) and using display solutions that allow the driver to virtually “see through” the A-pillar (Beresnev et al., 2018; Tragianis, 2014; Ylan, 2019). Blind spot monitoring systems (Forkenbrock et al., 2014) may also be worth considering.

Reducing A-pillar width would obviously reduce the risk associated with A-pillar blind spots. The consideration that the A-pillar also needs to be strong in order to protect the driver and passengers in case of roll-over, however, argues in favour of wider A-pillars (Bhise, 2016; Pipkorn et al., 2012). Thus, when making decisions about optimal A-pillar width, there is an inherent trade-off between competing safety concerns to consider. Accordingly, it is important to gain more precise knowledge of the relative risks and consequences involved. In light of the possible involvement of the illusion of absence, A-pillar obstruction may pose a more serious risk than one may intuitively expect, and it therefore appears important to direct more research effort towards clarifying the actual risk.

With regard to the question of optimal A-pillar width it should be emphasized, though, that for most drivers – namely those with vision on both eyes –, the ratio of the A-pillar width and the pupillary distance is of pivotal importance for visibility. As illustrated in Fig. 4, any other road user at any distance will be visible to at least one eye if this ratio is equal to or less than 1 (because all road users are wider than the interocular distance). Thus, reducing A-pillar width to a value corresponding to the pupillary distance is a particularly attractive target for visibility design. To what extent it is possible to engineer A-pillars as thin as that which also provide a reasonable roof stability is unknown to us. But if not, an alternative option that may be pursued would be to replace thick A-pillars with truss (grid) structures consisting of several thinner pillars which are each less wide than this critical value, such as in the Volvo SCC2—Safety Concept Car A-Pillar. This could conceivably provide the same level of stability as a single thick A-pillar while also providing much better visibility for drivers with vision on both eyes.

To summarize, in the present article, we have delineated how a recently described illusion of absence that plays a central role in the art of magic (Ekroll et al., 2017; Øhrn et al., 2019; Svalebjørg et al., 2020) may also be a contributing factor in traffic accidents. It would be premature to conclude that this is necessarily the case, but it appears plausible, and we believe that further research elucidating the potential role of the illusion of absence in road accidents may yield new insights with potentially important implications for road safety.

## Data Availability

Not applicable.

## Footnotes

1 By the same logic, magicians can also create the illusion that something *vanishes* into thin air by moving it *into* such a perceptually empty space created by the illusion of absence, but that is presumably less relevant for traffic accidents.

2 https://www.youtube.com/watch?v=QpvEmNKyg9A.

3 https://www.youtube.com/watch?v=sIQ_8bIco3s.

4 https://www.youtube.com/watch?v=SYeeTvitvFU.

5 The extrapolation may be filling-in of contours or surfaces or mere functional filling-in (grouping), as suggested by the study of Rensink and Enns (1998), which Wagemans (2018) has called “linking” (one of five types of perceptual grouping).

6 Movies that illustrative this are available in Sagberg and Sundfør (2020), as well as at http://www.insightlegalgraphics.com/portfolio-item/defense-pedestrian-blocked-by-a-pillar/ for an illustrative scenario.
